# Abstract screening using the automated tool Rayyan: results of effectiveness in three diagnostic test accuracy systematic reviews

**DOI:** 10.1186/s12874-022-01631-8

**Published:** 2022-06-02

**Authors:** Amir Valizadeh, Mana Moassefi, Amin Nakhostin-Ansari, Seyed Hossein Hosseini Asl, Mehrnush Saghab Torbati, Reyhaneh Aghajani, Zahra Maleki Ghorbani, Shahriar Faghani

**Affiliations:** 1 Neuroscience Institute, Tehran University of Medical Sciences, Tehran, Iran; 2 Sports Medicine Research Center, Neuroscience Institute, Tehran University of Medical Sciences, Tehran, Iran; 3 Students’ Scientific Research center, Exceptional Talents Development Center, Tehran University of Medical Sciences, Tehran, Iran; 4 Islamic Azad University of Zahedan, Zahedan, Iran; 5 Interdisciplinary Neuroscience Research Program (INRP), Tehran University of Medical Sciences, Tehran, Iran

**Keywords:** Rayyan, Abstract screening, Systematic reviews, Methodology

## Abstract

**Objective:**

To evaluate the performance of the automated abstract screening tool Rayyan.

**Methods:**

The records obtained from the search for three systematic reviews were manually screened in four stages. At the end of each stage, Rayyan was used to predict the eligibility score for the remaining records. At two different thresholds (≤2.5 and < 2.5 for exclusion of a record) Rayyan-generated ratings were compared with the decisions made by human reviewers in the manual screening process and the tool’s accuracy metrics were calculated.

**Results:**

Two thousand fifty-four records were screened manually, of which 379 were judged to be eligible for full-text assessment, and 112 were eventually included in the final review. For finding records eligible for full-text assessment, at the threshold of < 2.5 for exclusion, Rayyan managed to achieve sensitivity values of 97-99% with specificity values of 19-58%, while at the threshold of ≤2.5 for exclusion it had a specificity of 100% with sensitivity values of 1-29%. For the task of finding eligible reports for inclusion in the final review, almost similar results were obtained.

**Discussion:**

At the threshold of < 2.5 for exclusion, Rayyan managed to be a reliable tool for excluding ineligible records, but it was not much reliable for finding eligible records. We emphasize that this study was conducted on diagnostic test accuracy reviews, which are more difficult to screen due to inconsistent terminology.

**Supplementary Information:**

The online version contains supplementary material available at 10.1186/s12874-022-01631-8.

## Background

### Rationale

A systematic review (SR) is a scientific investigation that focuses on a specific question and uses explicit, prespecified scientific methods to identify, select, assess, and summarize the findings of similar but separate studies [1]. SRs are considered to have the strongest level of evidence (level 1) in modern evidence-based medicine [2]. As the body of scientific literature is rapidly growing, SRs are more appreciated by healthcare decision-makers, due to providing brief robust reports of new interventions and phenomena. Unfortunately, current methods for conducting SRs are very time-consuming, resulting in the slow production of these important scientific reports. In an analysis of 195 SRs in 2017 [3], the mean project length was 67.3 weeks with a range of 6–186 weeks. In the same analysis, the number of studies found in the literature searches ranged from 27 to 92,020 with a mean of 1781. As a rough conservative estimate, it is believed that titles and abstracts of search results could be screened at a rate of 60–120 per hour [3]. With some basic calculations applied to the mean value of 1781, it results in about 14.8 to 29.6 hours of exhaustive work for reviewers with a maximum range of 766.8 to 1533.6 hours. Taking into consideration that most organizations prefer this task to be done in duplicate by at least two masked reviewers to minimize the risk of bias in study selection, the above numbers could be doubled. These numbers indicate the significant amount of time and energy a team of authors has to spend just selecting the potentially eligible studies.

In recent years, a variety of automated tools have been introduced to facilitate the process of conduction of different parts of SRs, with different results. One of the main branches of these tools has been the study selection tools. Different automated tools have been developed for such tasks as Rayyan [4], Covidence [5], Abstrackr [6], Colandr [7], and EPPI-Reviewer [8]. These tools use text mining techniques to identify relevant information from text using statistical pattern learning that recognizes patterns in data. To achieve this, supervised learning algorithms are incorporated in their core, which tries to find patterns in the studies classified by the reviewers to predict the classification of unclassified records. These tools vary significantly in their core learning algorithm, offered features, and availability. In a scoping review in 2020 [9] Rayyan managed to get the highest score in weighted feature analysis and second place in the overall experience score (as rated by users in a survey) among these tools.

Rayyan, a web-based automated screening tool, developed by Qatar Computing Research Institute (QCRI) was initially launched in 2014 and is currently accessible at www.rayyan.ai. It uses text mining methods to facilitate semi-automatic screening of records for SRs. As a reviewer screens some records and labels them for either inclusion, exclusion, or “maybe” relevant to the subject of the review, the tool thrives for finding patterns and similarities to give a similarity score to each of the remaining records as a five-star rating. Higher ratings reflect the computed underlying probabilities of the record being included are higher, and vice versa. The simplicity of using Rayyan, combined with its completely free access, has made it quite popular among users. It also provides some interesting features such as allowing independent masked screening of the records by more than one user, creating custom labels for records, highlighting words for inclusion and exclusion (which significantly assists manual screening), and choosing the reason(s) for excluding a record. Rayyan’s code is written in the open-source framework Ruby on Rails [10] and runs on Heroku [11] which is a Platform as a Service based on the cloud-hosting Amazon Web Services. The Rayyan classification system is described in a paper [4] by the developers as follows:

Rayyan extracts all the words and pairs of words (bigrams) and MeSH terms following the removal of stop words and the stem of the remaining words from the titles/abstracts. These words are then used as features by the machine learning algorithm (support vector machine (SVM) classifier). As users label records as either excluded or included, the app uses the classifier to learn the features and build a model. The algorithm then runs on the records without a decision and gives a score to each of them, revealing how close it is to the include or exclude classes. That score is presented to the user as a five-star rating.

In the current paper, we aim to assess Rayyan’s effectiveness for screening title/abstract of records in three systematic reviews conducted by our team. It should be noted that the three reviews included in this study are diagnostic test accuracy (DTA) reviews. Due to inconsistent terminology, designing search strategies for DTA reviews is hard, resulting in a more difficult screening process as well.

### Terminology

In this paper, we used the standard terminology proposed by the PRISMA 2020 statement [12], with the addition of some new terms specific to this study:

- **Study:** An investigation, such as a clinical trial. A study might have multiple reports.
- **Report:** A document supplying information about a study. A report is typically a journal article or a preprint, but could also be a conference abstract, a dissertation, or a study register entry.
- **Record:** The title and abstract of a report indexed in a database. Records that refer to the same report are known as “duplicates”.
- **Record screening:** The process of screening records, also known as title/abstract screening.
- **Report screening:** The process of screening reports, also known as full-text assessment.
- **Eligible records:** Records that were judged to be eligible for report retrieval.
- Eligible reports: Reports that were judged to be eligible for inclusion in the final review.

### Objective

This study aims to evaluate the performance of the automated abstract screening tool Rayyan while screening records for three DTA systematic reviews. We intend to answer the following questions:

1. How precise was Rayyan in identifying eligible records following the manual screening of 20, 40, 60, and 80% of the records identified by the search for three DTA SRs?
2. How precise was Rayyan in identifying eligible reports following the manual screening of 20, 40, 60, and 80% of the records from the search results for three DTA SRs? It should be noted that Rayyan only evaluates records and not reports.

## Methods

This study’s design and methods are reported in line with the Standards for Reporting Diagnostic accuracy studies (STARD) checklist [13]. This study aims to evaluate the function of Rayyan in identifying eligible records and reports from the search results of three DTA SRs conducted in the Neuroscience Institute of Tehran University of Medical Sciences, Tehran, Iran. At the time of writing this paper, those SRs are still in the process of conduction. Their respective protocol has been published elsewhere [14].

### Study design

The three SRs were very similar in most aspects of the questions they were designed to answer. The only difference between studies was in the domain of the index test used. Eligibility criteria for the studies were as follows:

**Population:** patients with autism spectrum disorder (ASD) regardless of age, sex, and ethnicity.

**Index test:**

1. SR1: applied machine learning algorithms on cerebral structural magnetic resonance imaging (sMRI)
2. SR2: applied machine learning algorithms on cerebral resting-state functional magnetic resonance imaging (rs-fMRI)
3. SR3: applied machine learning algorithms on electroencephalogram (EEG)

**Target condition:** autism spectrum disorder (ASD) as defined by well-known diagnostic criteria (DSM-IV, DSM-V, ICD-11, ICD-10, ADOS, ADI-R, CARS, or GARS).

**Reference standard:** diagnosis made by a trained physician or psychologist.

**Study design:** cross-sectional design, including both single-gate (cohort type) and two-gates (case-control type) designs.

Search strategies were developed based on the above eligibility criteria, and the following databases were searched for relevant records: Embase, MEDLINE, APA PsycINFO, IEEE Xplore, Scopus, and Web of Science. We also searched grey literature through OpenGrey, Center for Research Libraries Online Catalogue (CRL), and Open Access Theses and Dissertations (OATD). Search strategies are presented in the Additional file 1.

Results of the search were imported into EndNote X9 [15], a citation management software. To avoid a redundant workload, duplicate records were removed using the EndNote deduplication system. The remaining records were exported and uploaded to the Rayyan web-based platform. Next, using Rayyan’s deduplication system, records with a similarity score of more than 0.85 were checked manually and removed if confirmed as duplicates. Thus, it must be considered that we only screened unique records. For the report screening process, we planned to discard records of the same report, however, all our eligible records were from unique reports.

### Test methods

**Fig. 1:**
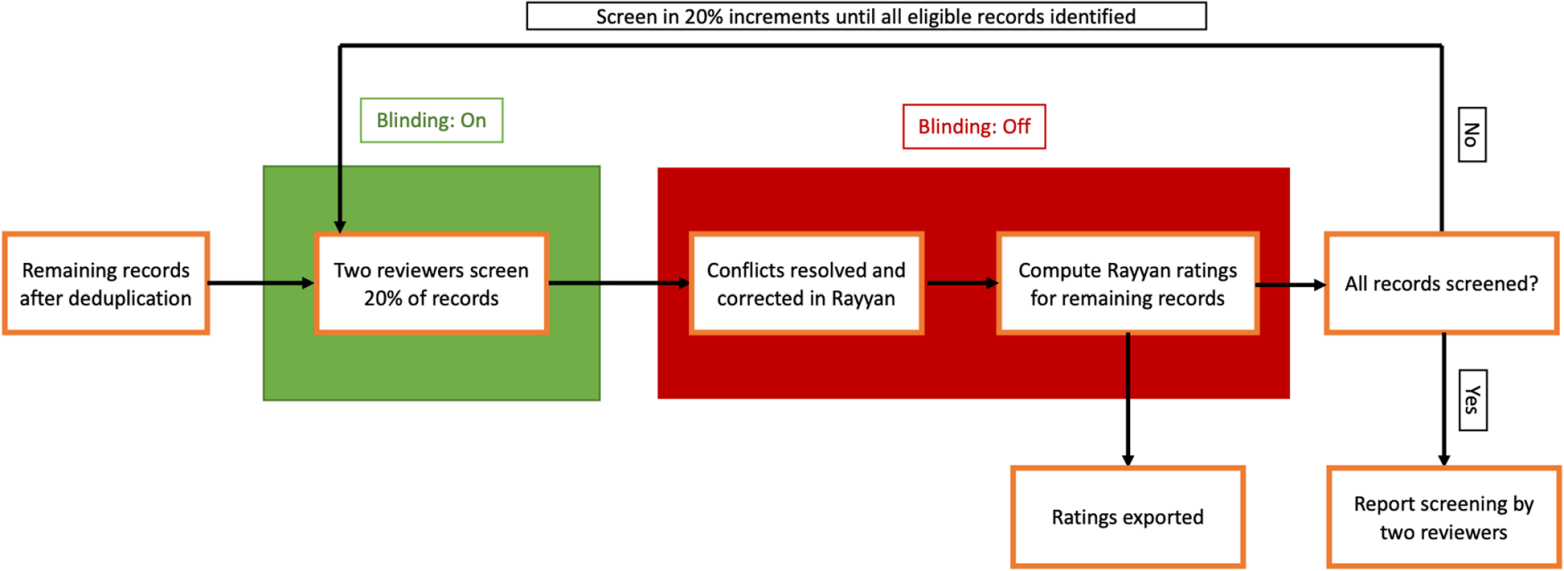
Summary of the screening process

In this study, the star ratings generated by Rayyan were the index test, and the human reviewers’ final decisions at the record screening stage were the reference standard. For each SR, two reviewers independently evaluated the first 20% (±0.1%) of the remaining records (following deduplication) in alphabetical order, labeling each as either “eligible”, “not eligible”, or “maybe eligible”. After the end of the independent screening of the first 20% (±0.1%) of records, the blinding feature of Rayyan was turned off and reviewers re-checked the conflicting decisions. Conflicts were resolved through discussion, and in case of disagreement, a third author was consulted. The third author also made the final decision for the “maybe eligible” records after careful evaluations, labeling each as either “eligible” or “not eligible”. After reaching a consensus, the reviewer with the decision that was different from the consensus result changed his/her submitted decision on the platform to match the consensus result. When all the conflicts were resolved, the “Compute Rating” feature of Rayyan was activated. This feature computes the ratings for the remaining records based on the patterns found in the decisions assigned to each screened record up to that point. All ratings were exported and saved in a file. Afterward, the blinding feature was turned back on and reviewers continued the record screening process for another 20% (±0.1%) of the records in alphabetical order. Although reviewers could see the computed ratings for the remaining records, they were strictly instructed to ignore them in making their judgments. The same process was taken in each step until all the records were screened and their assigned ratings were saved. Finally, the reports of the eligible records were retrieved and assessed independently by two reviewers for inclusion in the final review. A summary of the undertaken process is presented in Fig. 1.

### Analysis

Data were analyzed using R version 4.1 [16]. Rayyan assigns each record with one of the following ratings: 0.5 stars, 1.5 stars, 2.5 stars, 3.5 stars, or 4.5 stars. We chose two thresholds for our analyses: a rating of < 2.5 stars for exclusion (records with a rating of 0.5 or 1.5 are considered ineligible), and a rating of ≤2.5 stars for exclusion (records with a rating of 0.5, 1.5, or 2.5 are considered ineligible). These two thresholds were chosen because they were in the middle of the range of possible ratings, and thus, we hypothesized they might give the most balanced results for both sensitivity and specificity values. Additionally, we believe the decision to consider a record with a rating of 2.5, as eligible or ineligible, would be the hardest for a researcher, and thus, we aimed to report the diagnostic measures for the tool around this specific value.

Ratings were converted into a pair of binary dummy variables based on each threshold. By using this pair of variables, contingency tables were designed for each SR at each stage of the screening process for each threshold and each objective of the study. Then sensitivity (SEN), specificity (SPE), positive predictive value (PPV), negative predictive value (NPV), and F1 score for each stage and each objective of the study were calculated using the contingency tables. Considering that PPV and NPV are dependent on the ‘prevalence’ of studies that should be included in the review, which in turn depends on the sensitivity of the search strategy, we also calculated the point prevalence (PR) at each stage. SEN is the proportion of records that were judged to be “eligible” by Rayyan among all those that were eligible. On the other hand, PPV is the probability that when a record is judged to be “eligible” by Rayyan, that record is truly eligible. SPE is the proportion of records that were judged to be “not eligible” by Rayyan among all those that were not eligible, while NPV is the probability that when a record is judged to be “not eligible”, it is truly not eligible. Finally, the F1 score is a single number evaluation metric that is the harmonic mean of the precision (PPV) and recall (SEN). Given each contingency table, metrics were calculated based on the formulas presented in the Table 1.

**Table 1:** Formulas for calculated metrics. *FN* False-negative, *FP *False-positive, *NPV *Negative predictive value, *PPV *Positive predictive value, *SEN *Sensitivity, *SPE* Specificity, *TN* True-negative, *TP* True-positive

*TP = Number of eligible records (for objective 1) or eligible reports (for objective 2) identified by Rayyan eligible*				
*TN* = *Number of ineligible records* (*for objective* 1) *or einligible reports* (*for objective* 2) *identified by Rayyan ineligible*				
*FP* = *Number of ineligible records* (*for objective* 1) *or ineligible reports* (*for objective* 2) *identified by Rayyan as eligible*				
*FN* = *Number of eligible records* (*for objective* 1) *or eligible reports* (*for objective* 2) *identified by Rayyan as ineligible*				
$SEN=\frac{TP}{TP+ FN}$	$SPE=\frac{TN}{TN+ FP}$	$PPV=\frac{TP}{TP+ FP}$	$NPV=\frac{TN}{TN+ FN}$	$F1\ score=\frac{2 TP}{2 TP+ FP+ FN}$

Finally, all the calculated data were used to design line graphs to better represent the results.

## Results

### Flow of records

**Fig. 2:**
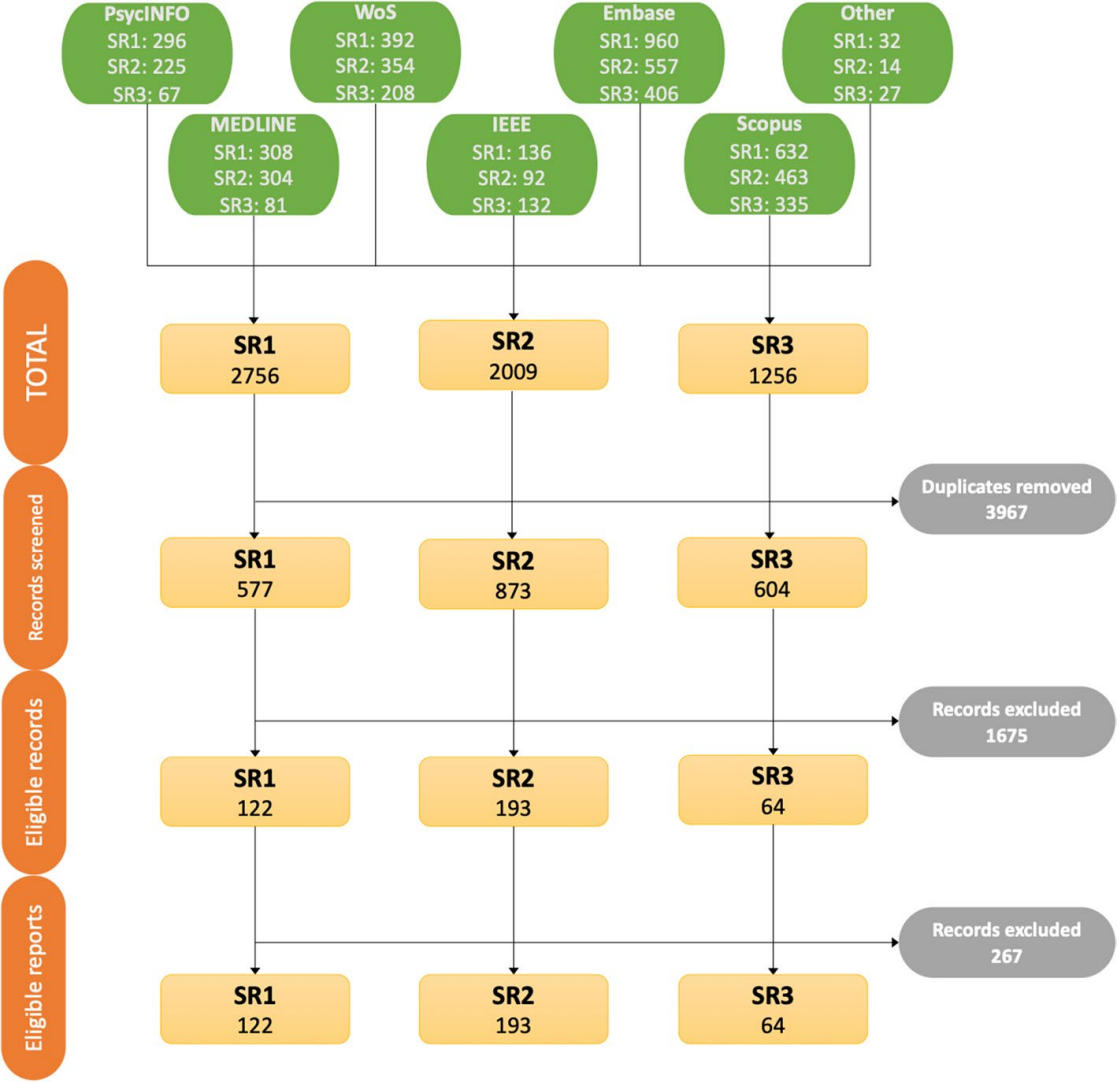
Flow of records

A total of 2054 records were screened manually, of which 379 (122 SR1, 193 SR2, and 64 SR3) were judged to be eligible records. Finally, 112 reports (25 SR1, 64 SR2, and 23 SR3) were included in the SRs following the report screening process. A summary of the flow of the records with the number of records assessed and discarded in each step is presented in Fig. 2.

### Test results

#### Identifying eligible records

**Fig. 3:**
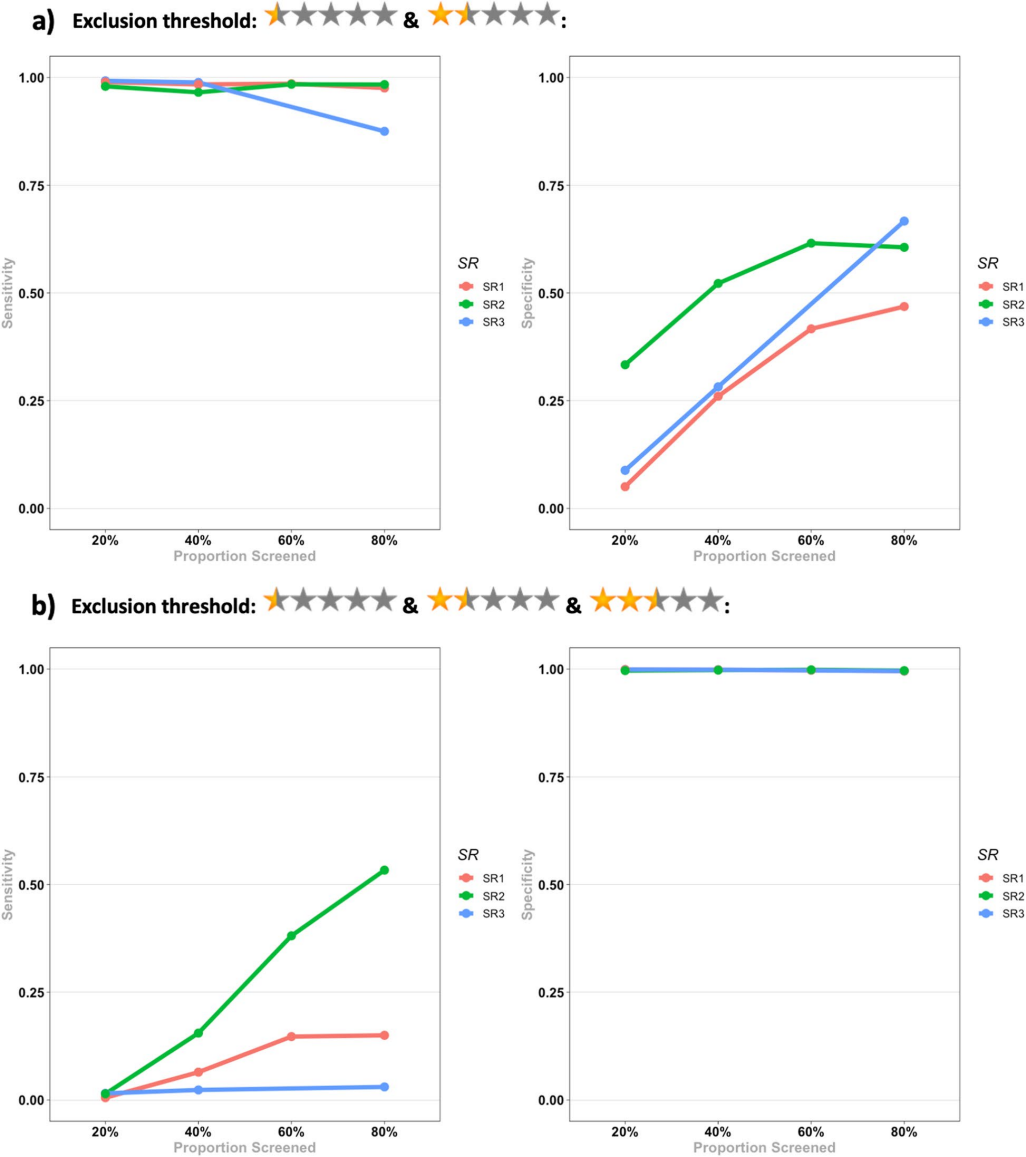
Evaluation metrics for the test accuracy for identifying eligible records for the 3 SRs in each screening stage for a) a threshold of less than 2.5 for exclusion; b) a threshold of 2.5 and less for exclusion. NPV: Negative predictive value, PPV: Positive predictive value

**Fig. 4:**
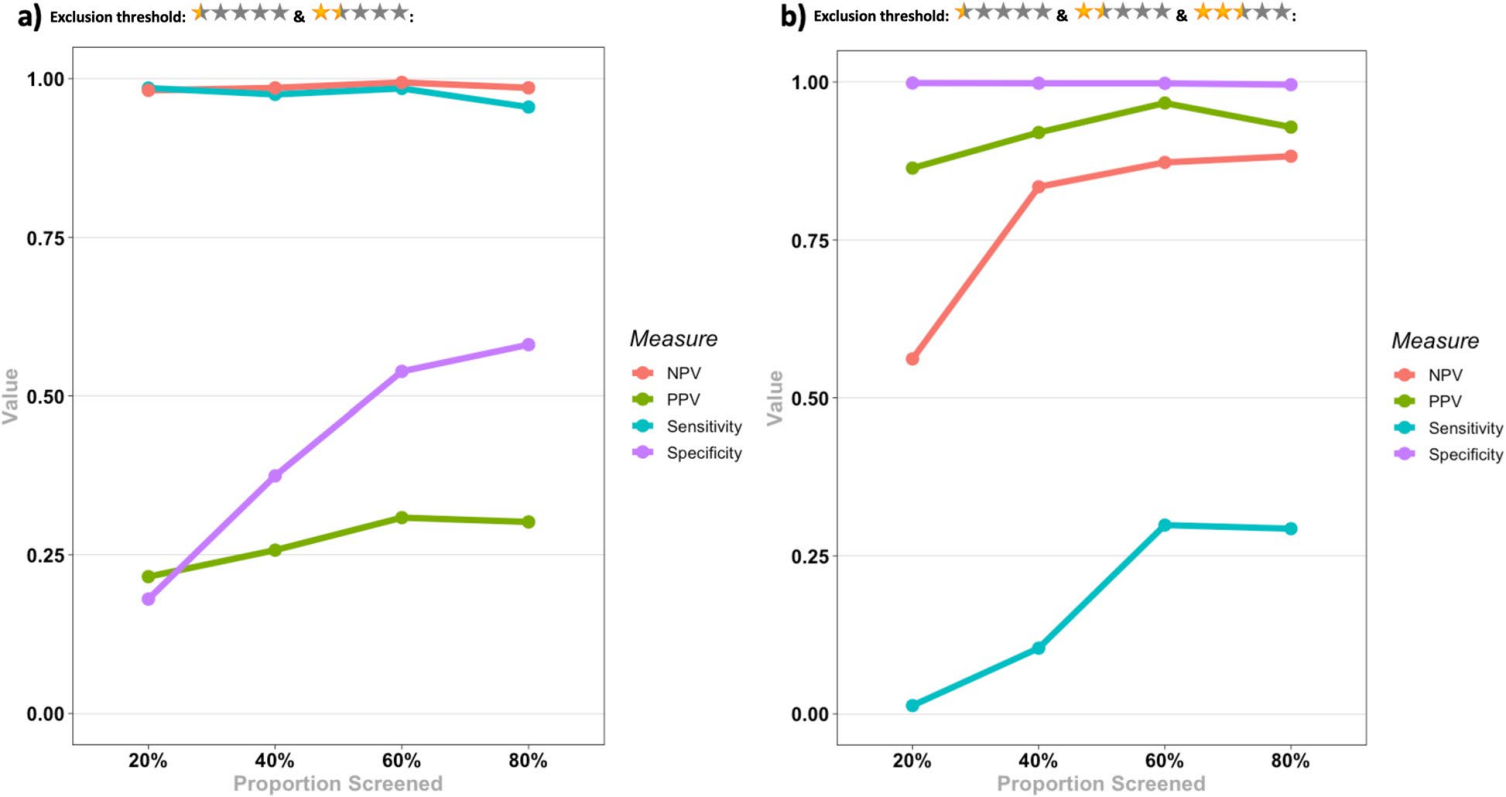
Evaluation metrics for the test accuracy for identifying eligible records for the 3 SRs in each screening stage for a) a threshold of less than 2.5 for exclusion; b) a threshold of 2.5 and less for exclusion. NPV: Negative predictive value, PPV: Positive predictive value

The results for the test accuracy for identifying eligible records for each SR and the pooled results are presented in Table 2, and Figs. 3 and 4.

**Table 2:**
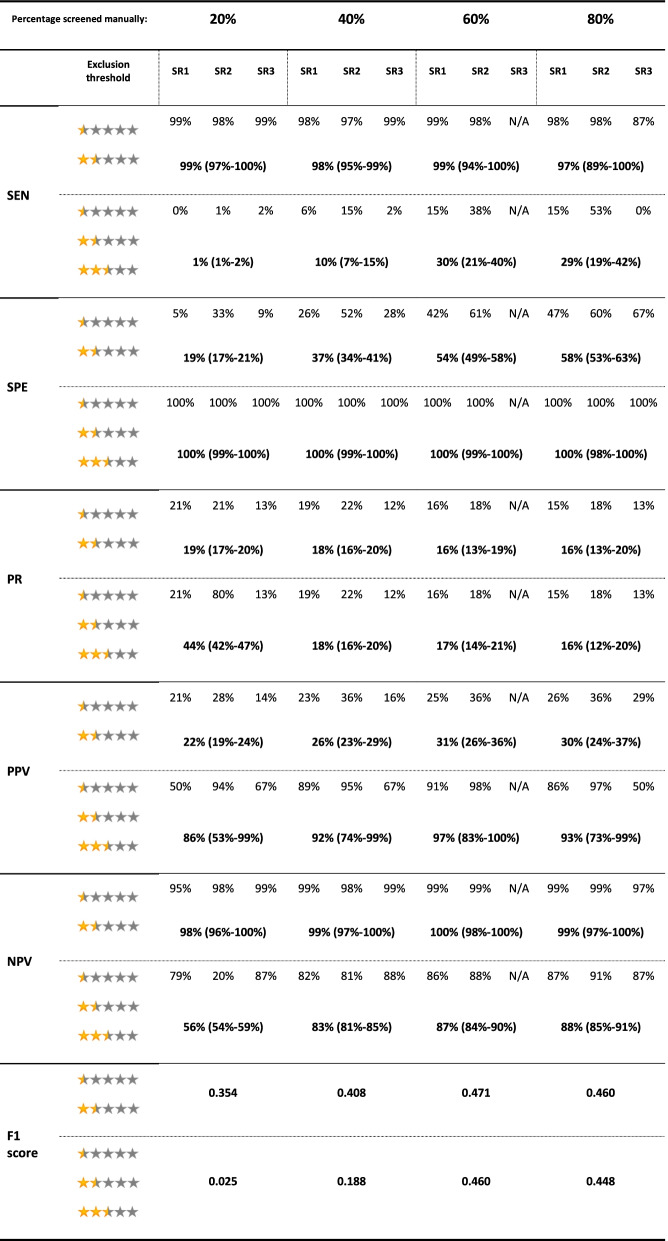
Evaluation metrics for the test accuracy for identifying eligible records for the 3 SRs in each screening stage. Pooled results for each metric in each stage are presented below the results of the three SRs. Numbers in the parentheses indicate 95% CI. *N/A *Not available, *NPV *Negative predictive value, *PPV *Positive predictive value, *PR* Prevalence, *SEN* Sensitivity, *SPE* Specificity

Considering a threshold of < 2.5 (0.5 and 1.5 stars) for exclusion of a record, Rayyan held a low PPV across all stages of screening, while it held almost a perfect NPV. A similar situation happened with SEN and SPE: SEN held an almost perfect value across all stages, while SPE managed to reach a maximum of 58% at the last stage of screening. Given these results, considering a threshold < 2.5 for exclusion, Rayyan managed to have an almost perfect exclusive function while having a relatively weak inclusive function, resulting in a suboptimal reduction of the workload.

Considering a threshold of ≤2.5 (0.5, 1.5, and 2.5 stars) for exclusion of a record, Rayyan had a perfect SPE while lacking in SEN (a maximum of 30%). The noticeable results were the PPV and NPV at this threshold. Even after the first stage of screening, it managed to reach a PPV of 86% (53-99%), while reaching a PPV of 92% (74-99%) after the second stage. It also managed to hold a relatively acceptable NPV after the first stage (56%), while reaching an NPV of 83% (81-85%) only after the second stage. Based on these results, Rayyan has the potential to reach acceptable PPV and NPV after manually screening 40% of records, considering a threshold of ≤2.5 for exclusion. It should be noted though that low SEN results for this threshold indicate the inappropriate exclusion of a considerable proportion of relevant records.

#### Identifying eligible reports

**Fig. 5:**
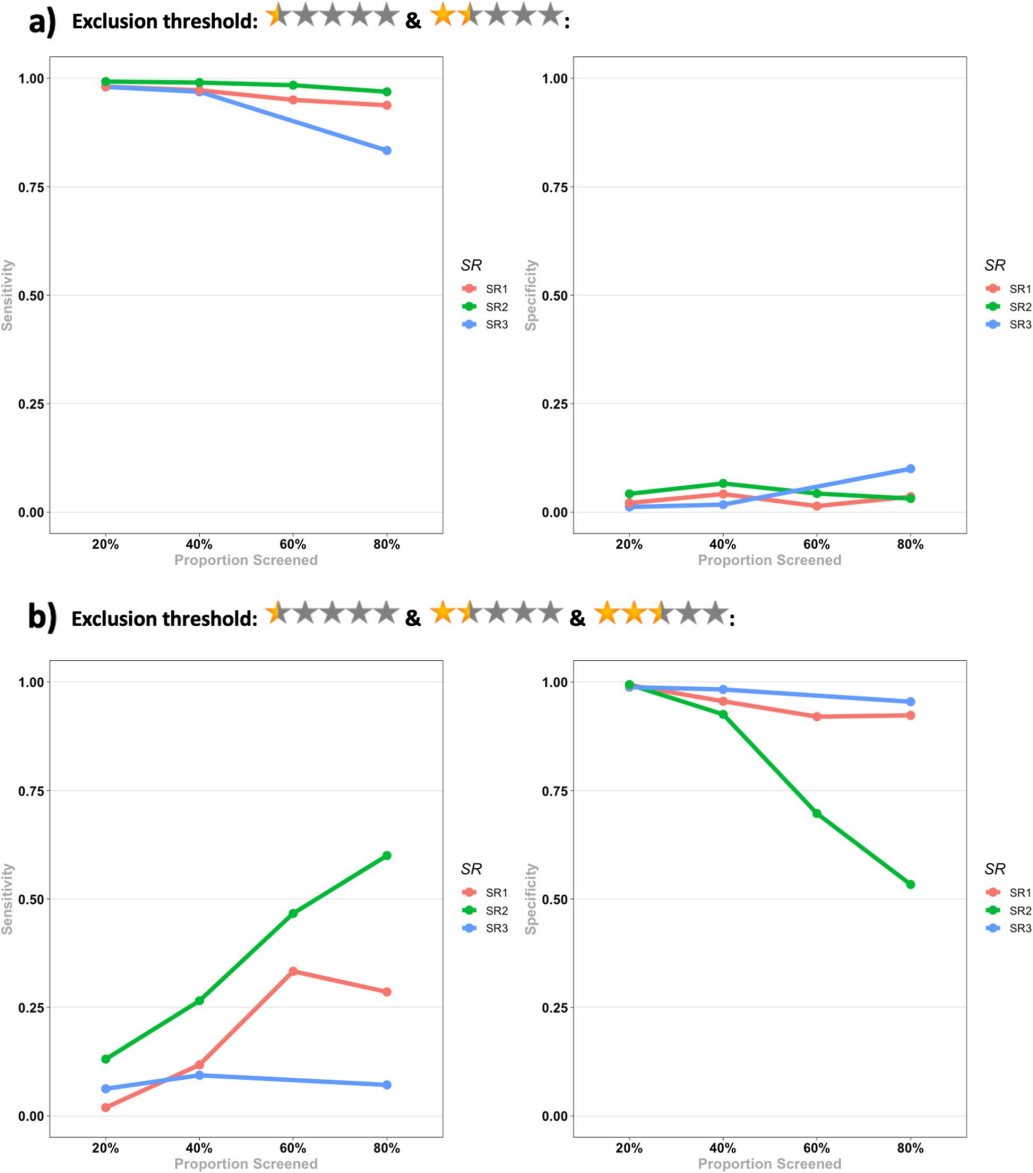
Evaluation metrics for the test accuracy for identifying eligible reports for the 3 SRs in each screening stage for **a**) a threshold of less than 2.5 for exclusion; **b**) a threshold of 2.5 and less for exclusion. NPV: Negative predictive value, PPV: Positive predictive value

**Fig. 6:**
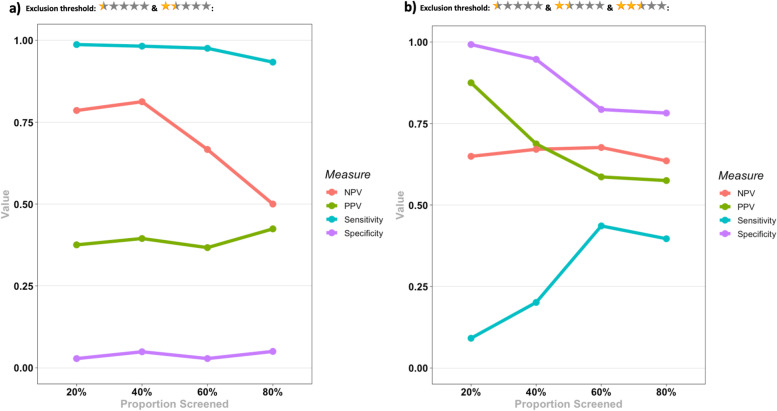
Evaluation metrics for the test accuracy for identifying eligible reports for the 3 SRs in each screening stage for **a**) a threshold of less than 2.5 for exclusion; **b**) a threshold of 2.5 and less for exclusion. NPV: Negative predictive value, PPV: Positive predictive value

The results for the test accuracy for identifying eligible reports for each SR and the pooled results are presented in Table 3, and Figs. 5 and 6.

**Table 3:**
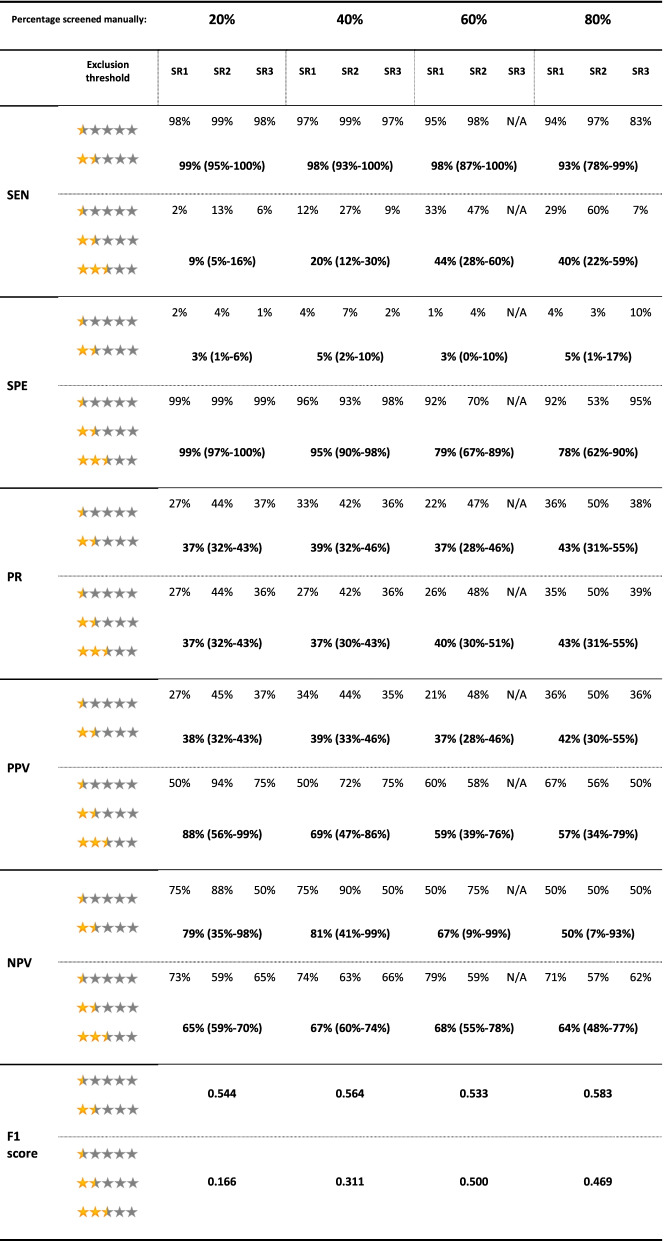
Evaluation metrics for the test accuracy for identifying eligible reports for the 3 SRs in each screening stage. Pooled results for each metric in each stage are presented below the results of the three SRs. Numbers in the parentheses indicate 95% CI. *N/A *Not available, *NPV *Negative predictive value, *PPV *Positive predictive value, *PR* Prevalence, *SEN* Sensitivity, *SPE* Specificity

Considering a threshold of < 2.5 (0.5 and 1.5 stars) for exclusion of a report, Rayyan held an almost perfect SEN across all stages, while SPE was very poor (3-5%). PPV was in the range of 37-43% across the stages, but NPV values were higher, reaching a maximum of 81%. Given these results, considering a threshold of < 2.5 for exclusion, Rayyan managed to have an almost perfect exclusive function while having a very weak inclusive function.

Considering a threshold of ≤2.5 (0.5, 1.5, and 2.5 stars) for exclusion of a report, Rayyan had high SPE values (78-99%) while having relatively low SEN values (a maximum of 44%). PPV was in the range of 57-88% across all stages, while NPV was in the range of 64-68%. The noticeable result was that NPV values remain consistent across all stages. Based on these results, Rayyan had almost balanced PPV and NPV values considering a threshold of ≤2.5 for exclusion, although low SEN values for this threshold indicate that a considerable proportion of relevant reports may be excluded by mistake.

## Discussion

### Summary of main findings

A summary of the main results of this study is presented in Table 4.

**Table 4:**
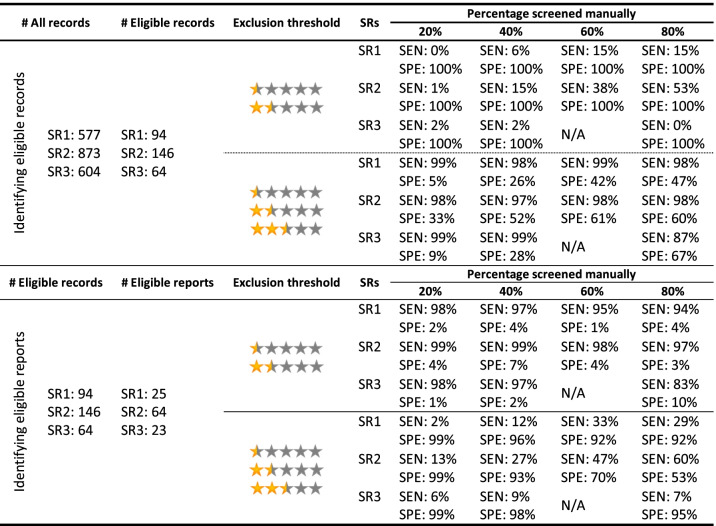
Summary of main findings. *N/A *Not available, *SEN *Sensitivity, *SPE *Specificity

### Relevant studies

For a brief review of previous relevant studies, check Table 5.

**Table 5:** Summary of the relevant studies. FNR: False-negative rate. *SEN *Sensitivity, *SPE *Specificity

**Identifying eligible records**					
**Study ID**	**Tool**	**Studies**	**All records**	**Results**	**Comments**
Olofsson 2017 [16]	Rayyan [4]	3 SRs and 3 literature reviews	7956	SEN of 21-88% after screening 25% of records. SEN of 86-98% after screening 50% of records. SEN of 89-100% after screening 75% of records.	Thresholds used were not reported. SPE rates were not reported.
Rathbone 2015 [17]	Abstrackr [6]	4 SRs	SR1: 1415 SR2: 517 SR3: 1735 SR4: 1042	SR1: Precision of 16.8% and FNR of 10% after screening 18% of records. SR2: Precision of 24.7% and FNR of 14.5% after screening 23% of records. SR3: Precision of 29.2% and FNR of 4.7% after screening 7% of records. SR4: Precision of 45.5% and FNR of 2.4% after screening 12% of records.	SEN and SPE rates were not reported.
Gates 2018 [18]	Abstrackr [6]	3 SRs and 1 descriptive analysis (DA)	SR1: 12763 SR2: 5893 SR3: 47385 DA: 5243	SR1: SPE of 69% and SEN of 79% after screening 2.2% of records. SR2: SPE of 85% and SEN of 92% after screening 10.3% of records. SR3: SPE of 90% and SEN of 82% after screening 0.7% of records. DA: SPE of 19% and SEN of 96% after screening 4% of records.	–
Tsou 2020 [19]	Abstrackr [6] and EPPI-Reviewer [8]	9 SRs	SR1: 9038 SR2: 3181 SR3: 2706 SR4: 889 SR5: 673 SR6: 651 SR7: 500 SR8: 427 SR9: 226	For Abstrackr, SEN of 100% after screening 71.1, 51.5, 96, 95.6, 99, 85.9, 88.2, 99.3, and 93.8% of records for SR1 to SR9 respectively. For EPPI-Reviewer, SEN of 100% after screening 61.7, 39.9, 91.3, 94.6, 97.9, 86.3, 88.2, 98.8, and 91.6% of records for SR1 to SR9 respectively.	They also reported diagnostic metrics for identifying eligible reports.
Chai 2021 [20]	Research Screener [20]	9 SRs and 2 scoping reviews (SCR)	SR1: 813 SR2: 2249 SR3: 2584 SR4: 368 SR5: 870 SR6: 306 SR7: 23423 SR8: 13376 SR9: 1686 SCR1: 16506 SCR2: 1230	SEN of 100% after screening 32, 13, 6, 5, 4, 4, 5, 13, and 14% of records for SR1 to SR9 respectively. SEN of 100% after screening 40 and 38% of records for SCR1 to SCR2 respectively.	This tool utilizes deep learning algorithms.
**Identifying eligible reports**					
**Study ID**	**Tool**	**Studies**	**Eligible records**	**Results**	
Tsou 2020 [19]	Abstrackr [6] and EPPI-Reviewer [8]	9 SRs	SR1: 696 SR2: 200 SR3: 843 SR4: 107 SR5: 267 SR6: 73 SR7: 166 SR8: 149 SR9: 104	For Abstrackr, SEN of 100% after screening 40.7, 39.8, 81.2, 100, 71.6, 56.4, 41.2, 60, and 71.2% of eligible records for SR1 to SR9 respectively. For EPPI-Reviewer, SEN of 100% after screening 41, 39.8, 97.1, 70, 74, 30.1, 31.8, 59.4, and 51.3% of eligible records for SR1 to SR9 respectively.	

A previous study on Rayyan [4] by Olofsson et al. in 2017 [17] revealed promising results for the effectiveness of the tool for identifying eligible records of six reviews (3 SRs and 3 literature reviews). In their study, 21 to 88% of eligible records were identified by the time the first quarter had been screened, 86 to 98% when half were screened, and 89-100% when three quarters were screened. Their study did not mention the threshold used for their results.

In a study by Rathbone et al. in 2015 [18] on Abstrackr [6], they reported precisions of 16.8 to 45.5% and false-negative rates of 2.4 to 14.5% for identifying eligible records after screening less than 18% of records for 4 reviews. Sensitivity and specificity rates were not reported.

Gates et al. [19] conducted another study on Abstrackr in 2018 and reported sensitivity and specificity rates of 79-96% and 19-90% for identifying eligible records after screening 0.7-10.3% of records for 3 SRs and 1 descriptive analysis study.

In 2020, Tsou et al. [20] compared the effectiveness of Abstrackr and EPPI-Reviewer [8] for the semi-automated screening of records of 9 SRs. They reported better results with the EPPI-Reviewer, achieving a sensitivity of 100% for identifying eligible records after manually screening 39.9-89.8% of records. They also evaluated the effectiveness of those tools for identifying eligible reports. For the EPPI-Reviewer, they achieved a sensitivity of 100% after manually screening 30.1-97.1% of records, while for Abstrackr, they achieved the same sensitivity value after manually screening 39.8-100% of records.

Chai et al. in 2021 [21] introduced a new tool named “Research Screener” which utilizes deep learning algorithms for the semi-automated screening process. In their validation study on 9 SRs, a sensitivity rate of 100% for identifying eligible records was achieved after manually screening only 4-32% of records.

### Interpretation of the results

This study aimed to evaluate the performance of Rayyan, a tool for the semi-automatic screening of records. Here, we reported two sets of results: considering a rating of < 2.5 (0.5 and 1.5) for exclusion and considering a rating of ≤2.5 (0.5, 1.5, and 2.5) for exclusion. We believe there was no need for analyses on other thresholds because our results indicate the presence of a huge difference in the sensitivity and specificity of the tool around these two close thresholds. Thus, it is predictable that a higher threshold would only result in a drop in sensitivity (without a considerable change in specificity), and lower thresholds only decrease specificity without much of a change in the sensitivity.

Around the thresholds used in our study, we see very different results. For the task of identifying eligible records, at the threshold of ≤2.5 (0.5, 1.5, and 2.5) for exclusion, we achieved specificity rates of 100% after just screening 20% of the records, while at the threshold of < 2.5 (0.5 and 1.5) for exclusion, we achieved sensitivity rates of 98-99% following the manual screening of a similar proportion of the records, which is close to the results of the study of Olofsson et al. [17] (sensitivity of 21-88% after screening of 25% of the records and 86-98% after screening 50% of the records). Such contradictory results around these two close thresholds are an indication of the poor differentiation ability of the tool. In contrast, the study of Gates et al. [19] on Abstrackr [6] for 3 SRs achieved both good sensitivity and specificity results after manually screening a similar number of records (69-90% and 79-92% respectively). On the other hand, the study of Tsou et al. [20] reported that Abstrackr reached a sensitivity of 100% after screening a greater number of records (51-99% of the records in 9 SRs), compared to our results on Rayyan. Their study did not report specificity rates.

For the task of identifying eligible reports, sensitivity values followed a similar pattern to those found in the task of identifying eligible records, but specificity values were substantially different. At the threshold of ≤2.5 (0.5, 1.5, and 2.5) for exclusion, two SRs maintained high specificity values, while the third SR had a significant drop in specificity following each stage of the screening. On the other hand, at the threshold of < 2.5 (0.5 and 1.5) for exclusion, results showed very poor specificity values for all three SRs. Compared to the results of the study of Tsou et al. [20], our results indicate that Rayyan might have a sensitivity superior to Abstrackr at the threshold of < 2.5 (0.5 and 1.5) for exclusion, but it is not possible to compare the specificity of the tools as they did not report this metric.

Despite all that, the question is which threshold should be considered as the optimal choice? Noticing that one of the main privileges of using an automated screening tool should be reducing workload, it is of great importance for the tool to reach an appropriate level of learning as fast as possible. Taking that into consideration, it seems that a threshold of < 2.5 (0.5 and 1.5) for exclusion is the optimal choice for record screening, as it achieved a good F1 score (0.354) with just 20% of the records manually screened. Similar results were observed for the task of report screening at this threshold, where Rayyan achieved an F1 score of 0.544 with just 20% of the records manually screened. As stated in the handbook of Cochrane [3], when searching for and selecting studies, reviewers should use methods that aim for “maximized” sensitivity whilst striving for “reasonable” precision. This threshold indeed showed very high sensitivity in our results. On the other hand, specificity was very low in this threshold (5-33% for finding eligible records after manually screening 20% of records and 26-52% after manually screening 40% of records, and a maximum of 10% for finding eligible reports), which implies the inclusive function of the tool is not reliable at this threshold. Nevertheless, as sensitivity should be prioritized above specificity in the selection of records, this threshold is deemed the optimal choice, because it achieves “maximized” sensitivity while holding to the highest possible specificity at such great sensitivity rates. In rare cases when specificity comes first (for example when the time resources are limited for conducting an SR), a threshold of ≤2.5 (0.5, 1.5, and 2.5) for exclusion could be the optimal choice for finding eligible records and reports. Although when interpreting these results, it should also be considered that our 3 SRs were DTA reviews on machine learning algorithms. Both DTA and machine learning algorithm studies are very difficult to screen, because of inconsistent terminology.

Considering that this tool utilizes machine learning algorithms at its core, it also suffers the same issues. One of these issues is the class imbalance problem. Data are said to suffer the class imbalance problem when the class distributions are highly imbalanced. In this context, many classification learning algorithms have low predictive accuracy for the infrequent class [22]. In our study, 379 of 2054 records were judged to be eligible records, only 18.5% of the data, while only 112 were judged to be eligible reports (5.4% of the data). Such a significant imbalance could have strongly affected the training process of the learning algorithm. Developers of the tool are recommended to use cost-sensitive learning techniques [23] in future updates to tackle this issue.

Overall, knowing that the algorithm used as the core of Rayyan (SVM) is not considered the optimal classification algorithm in the era of deep learning, our results were not much of a surprise. Although developers did not specify the kernel used by the SVM in Rayyan, it is most possible that it only utilizes a linear kernel, which is incapable of learning the complex non-linear relationships in the data. Knowing that such an algorithm does not require extensive computational resources, it might be a good choice for a free app at the time of initial release, but considering the advances in computer hardware products in recent years, it may be possible to utilize a more advanced classification algorithm given the same expenses. Research Screener [21] is a new tool that utilizes deep learning algorithms and performs record screening via learning text embeddings. Although this tool is, at the moment of writing this paper, being tested in closed beta trials. In the validation study [21] published by the developers of the app, it managed to reach a sensitivity of 100% after 4-32% of the records were manually screened in 9 SRs. Unfortunately, specificity results were not reported.

### Limitations

First, it should be noted that our study included three SRs of the same review type, DTA reviews. Designing specific search strategies for these kinds of reviews is difficult (due to inconsistent terminology) which makes screening often more difficult as well, compared to reviews on interventions that mostly include randomized controlled trials.

Also, the reviewers could see the ratings computed by the platform in each screening stage. Although we instructed them to ignore these ratings in their judgments, some risks of bias might still exist.

It should also be noted that we only assessed one outcome in our study (diagnostic accuracy measures). Other studies on Rayyan and other similar tools did also evaluate other outcomes such as workload savings [18, 19], users’ satisfaction and recommendations [17], and diagnostic accuracy of the tool for large and small SRs seperately [20].

Another important issue in our study that requires special consideration is the complex nature of the index test of the SRs. All the index tests consisted of two components that may have resulted in lower evaluation metrics: a neural response recording technique (sMRI, rs-fMRI, and EEG) and a machine learning algorithm (which consists of many different terms).

Another limitation was that the terminology of machine learning and statistics have many similar words, which may have also caused bias in the results. For example, the word “regression” could mean either a statistical method or a machine learning algorithm. Also, considering that the three SRs included in this study had similar topics, it further reduces the generalizability power of our results.

Finally, data for the 3rd stage of study selection in the SR of EEG was missing because unfortunately, the results for that stage of screening were accidentally lost. It could have potentially affected our results.

### Implications for practice

Considering that our study was on DTA SRs of machine learning studies, inconsistent terminology is believed to have a huge impact on our results. With that being said, we still managed to achieve almost perfect sensitivity values for finding eligible records and reports at the threshold < 2.5 (0.5 and 1.5) for exclusion after manually screening only 20% of the records. Such considerable exclusive power can greatly help the production of SRs by reducing the workload significantly. This exclusive accuracy can also come in handy in conducting live SRs where screening hundreds of records might be necessary at frequent short time intervals. In exceptional circumstances when review resources are scarce and specificity rates are the priority, a threshold of ≤2.5 (0.5, 1.5, and 2.5) for exclusion can be used to achieve reliable results for the screening process rapidly, though the exclusion of a proportion of relevant records is expected.

### Implications for research

Future research on semi-automated records screening tools should consider some issues. First, diagnostic measures should be reported appropriately. We noticed that most of the relevant studies only reported one or two metrics, mostly just sensitivity values, while other measures are also required for an in-depth evaluation of the tool. We also recommend including other outcomes than just the diagnostic measures, such as users’ satisfaction, ease of use, workload saving, and possible critics and recommendations of the users. Reporting results after smaller proportions of manual screening (e.g., 10, 20%, etc.) is also encouraged. We also strongly suggest the evaluation of screening tools that utilize modern deep learning methods when they become available, such as Research Screener [21]. Finally, for a more informative design, we suggest future research to compare the decisions of one reviewer and the record screening tool against an additional reviewer pair without the record screening tool, in which case it is possible to find the potential cases where reviewers missed eligible records.

## Supplementary Information

**Additional file 1.**

## Data Availability

The datasets used and analyzed during the current study are available from the corresponding author on request.
