# Supplementary material for: Abstract screening using the automated tool Rayyan: results of effectiveness in three diagnostic test accuracy systematic reviews
Source: BMC Med Res Methodol. 2022 Jun 2;22:160. doi: 10.1186/s12874-022-01631-8 (PMC9161508; doi:10.1186/s12874-022-01631-8)
Supplement: Supplementary file 1 — Additional file 1. [file 12874_2022_1631_MOESM1_ESM.pdf]

Appendix

s-MRI review

**Database** <date>	**Search syntax**
**Embase** Elsevier <1974 to 05.22.2021>	#1 ‘autism’/exp<br>#2 'asperger syndrom*':ab,ti,kw OR 'autis*':ab,ti,kw OR 'child* development* disorder*':ab,ti,kw<br>#3 #1 OR #2<br>#4 ‘nuclear magnetic resonance imaging’/exp<br>#5 mri:ab,ti,kw OR 'magnetic resonance imaging':ab,ti,kw OR smri:ab,ti,kw OR 's mri':ab,ti,kw<br>#6 #4 OR #5<br>#7 ‘information processing’/exp<br>#8 'artificial intelligence*':ab,ti,kw OR 'deep learning*':ab,ti,kw OR 'machine learning*':ab,ti,kw OR 'machine intelligence*':ab,ti,kw OR 'supervised learning*':ab,ti,kw OR 'unsupervised learning*':ab,ti,kw OR ‘semi-supervised learning*’:ab,ti,kw OR ‘reinforcement learning*’:ab,ti,kw OR 'regression algorithm*':ab,ti,kw OR 'learning vector quantization*':ab,ti,kw OR 'self organizing map*':ab,ti,kw OR 'regularization algorithm*':ab,ti,kw OR 'iterative dichotomiser*':ab,ti,kw OR 'decision stump*':ab,ti,kw OR 'clustering algorithm*':ab,ti,kw OR 'k-means*':ab,ti,kw OR 'k-medians*':ab,ti,kw OR 'hierarchical clustering*':ab,ti,kw OR 'apriori*':ab,ti,kw OR 'auto encoder*':ab,ti,kw OR 'auto-encoder*':ab,ti,kw OR 'markov*':ab,ti,kw OR 'classification algorithm*':ab,ti,kw OR 'locally weighted learning*':ab,ti,kw OR 'support vector machine*':ab,ti,kw OR 'svm':ab,ti,kw OR 'decision tree*':ab,ti,kw OR 'component analy*':ab,ti,kw OR 'pca':ab,ti,kw OR 'ica':ab,ti,kw OR 'neural network*':ab,ti,kw OR 'hierarchical learning*':ab,ti,kw OR 'latent class analy*':ab,ti,kw OR 'latent class model*':ab,ti,kw OR 'latent variable model*':ab,ti,kw OR 'boltzmann':ab,ti,kw OR 'deep belief network*':ab,ti,kw OR ‘lasso regression*':ab,ti,kw OR 'lda':ab,ti,kw OR 'adtree':ab,ti,kw OR ‘ridge regression':ab,ti,kw OR 'enet':ab,ti,kw OR ‘fnn':ab,ti,kw OR ‘firefly algorithm*':ab,ti,kw OR ‘multivariate adaptive regression splines*’:ab,ti,kw OR ‘locally estimated scatterplot smoothing*’:ab,ti,kw OR ‘instance base*’:ab,ti,kw OR ‘nearest neighbor*’:ab,ti,kw OR ‘regularization algorithm*’:ab,ti,kw OR ‘least absolute shrinkage select*’:ab,ti,kw OR ‘least angle regression*’:ab,ti,kw OR ‘eclat*’:ab,ti,kw OR ‘back propagation*’:ab,ti,kw OR ‘hopfield network*’:ab,ti,kw OR ‘radial basis function network*’:ab,ti,kw OR ‘dimensionality reduction algorithm*’:ab,ti,kw OR ‘ensemble algorithm*’:ab,ti,kw OR ‘adaboost’:ab,ti,kw OR ‘stacked generalization*’:ab,ti,kw OR ‘relu’:ab,ti,kw OR ‘naive bayes’:ab,ti,kw<br>#9 #7 OR #8<br>#10 #3 AND #6 AND #9
**MEDLINE** OvidSP <1996 to 05.22.2021>	1 exp Autism Spectrum Disorder/<br>2 (asperger syndrom* or autis* or child* development* disorder*).mp.<br>3 1 or 2<br>4 exp Magnetic Resonance Imaging/<br>5 (mri or magnetic resonance imaging or smri or s-mri or structural-mri).mp.<br>6 4 or 5<br>7 exp Algorithms/ or exp Neural Networks, Computer/ or exp Decision Trees/<br>8 (artificial intelligence* or deep learning* or machine learning* or machine intelligence* or supervised learning* or unsupervised learning* or semi-supervised learning* or reinforcement learning* or regression algorithm* or learning vector quantization* or self organizing map* or regularization

	algorithm* or iterative dichotomiser* or decision stump* or clustering algorithm* or k-means* or k-medians* or hierarchical clustering* or apriori* or auto encoder* or auto-encoder* or markov* or classification algorithm* or locally weighted learning* or support vector machine* or svm or decision tree* or component analy* or pca or ica or neural network* or hierarchical learning* or latent class analy* or latent class model* or latent variable model* or boltzmann or deep belief network* or lasso regression* or lda or adtree or ridge regression or enet or fnn or firefly algorithm* or multivariate adaptive regression splines* or locally estimated scatterplot smoothing* or instance base* or nearest neighbor* or regularization algorithm* or least absolute shrinkage select* or least angle regression* or eclat* or back propagation* or hopfield network* or radial basis function network* or dimensionality reduction algorithm* or ensemble algorithm* or adaboost or stacked generalization* or relu or naive bayes).mp.<br>9 7 or 8<br>10 3 and 6 and 9
**APA PsycINFO**<br>OvidSP<br><1987 to 05.22.2021>	1 exp Autism Spectrum Disorders/<br>2 (asperger syndrom* or autis* or child* development* disorder*).mp.<br>3 1 or 2<br>4 exp Magnetic Resonance Imaging/<br>5 (mri or magnetic resonance imaging or smri or s-mri or structural-mri).mp.<br>6 4 or 5<br>7 exp Artificial Intelligence/<br>8 (artificial intelligence* or deep learning* or machine learning* or machine intelligence* or supervised learning* or unsupervised learning* or semi-supervised learning* or reinforcement learning* or regression algorithm* or learning vector quantization* or self organizing map* or regularization algorithm* or iterative dichotomiser* or decision stump* or clustering algorithm* or k-means* or k-medians* or hierarchical clustering* or apriori* or auto encoder* or auto-encoder* or markov* or classification algorithm* or locally weighted learning* or support vector machine* or svm or decision tree* or component analy* or pca or ica or neural network* or hierarchical learning* or latent class analy* or latent class model* or latent variable model* or boltzmann or deep belief network* or lasso regression* or lda or adtree or ridge regression or enet or fnn or firefly algorithm* or multivariate adaptive regression splines* or locally estimated scatterplot smoothing* or instance base* or nearest neighbor* or regularization algorithm* or least absolute shrinkage select* or least angle regression* or eclat* or back propagation* or hopfield network* or radial basis function network* or dimensionality reduction algorithm* or ensemble algorithm* or adaboost or stacked generalization* or relu or naive bayes).mp.<br>9 7 or 8<br>10 3 and 6 and 9
**IEEE Xplore**<br><1988 to 05.22.2021>	(asperger syndrome OR autis* OR child development disorder) AND (mri OR magnetic resonance imaging OR smri OR s-mri OR structural-mri) AND (artificial intelligence OR deep learning OR machine learning OR machine intelligence OR supervised learning OR unsupervised learning OR semi-supervised learning OR reinforcement learning OR regression algorithm OR learning vector quantization OR self organizing map OR regularization algorithm OR iterative dichotomiser OR decision stump OR clustering algorithm OR k-means OR k-medians OR hierarchical clustering OR apriori OR auto encoder OR auto-encoder OR markov OR classification algorithm OR locally weighted learning OR support vector machine OR svm OR decision tree* OR component analysis OR pca OR ica OR neural network* OR hierarchical learning OR latent class analysis OR latent class model OR latent variable model OR boltzmann OR deep belief network OR lasso regression OR lda OR adtree OR ridge regression OR enet OR fnn OR firefly algorithm OR multivariate adaptive regression splines OR locally estimated scatterplot smoothing OR instance base OR nearest neighbors OR regularization algorithm OR least absolute shrinkage selection OR least angle regression OR eclat OR back propagation OR hopfield network OR radial basis function network OR dimensionality reduction algorithm OR ensemble algorithm OR adaboost OR stacked generalization OR relu OR naive bayes)
**Scopus**<br>Elsevier<br><1996 to 05.22.2021>	TITLE-ABS-KEY(“asperger syndrome*” OR “autis*” OR “child development disorder*”) AND TITLE-ABS-KEY(“mri” OR “magnetic resonance imaging” OR “smri” OR “s-mri” OR “structural-mri”) AND TITLE-ABS-KEY(“artificial intelligence*” OR “deep learning*” OR “machine learning*” OR “machine intelligence*” OR “supervised learning*” OR “unsupervised learning*” OR “semi-supervised learning*” OR “reinforcement learning*” OR “regression algorithm*” OR “learning vector quantization*” OR “self organizing map*” OR “regularization algorithm*” OR “iterative dichotomiser*” OR “decision stump*” OR “clustering algorithm*” OR “k-means” OR “k-medians” OR “hierarchical clustering*” OR “apriori” OR “auto encoder*” OR “auto-encoder*” OR “markov” OR

	“classification algorithm*” OR “locally weighted learning*” OR “support vector machine*” OR “svm” OR ”decision tree*” OR “component analy*” OR “pca” OR “ica” OR “neural network*” OR “hierarchical learning*” OR “latent class analy*” OR “latent class model*” OR “latent variable model*” OR “boltzmann” OR “deep belief network*” OR “lasso regression*” OR “lda” OR “adtree*” OR “ridge regression*” OR “enet” OR “fnn” OR “firefly algorithm*” OR “multivariate adaptive regression splines*” OR “locally estimated scatterplot smoothing*” OR “instance base*” OR “instance-base*” OR “nearest neighbor*” OR “regularization algorithm*” OR “least absolute shrinkage select*” OR “least angle regression*” OR “eclat” OR “back propagation*” OR “hopfield network*” OR “radial basis function network*” OR “dimensionality reduction algorithm*” OR “ensemble algorithm*” OR “adaboost” OR “stacked generalization*” OR “relu” OR “naive bayes*”)
**Web of Science** Core Collection <1900 to 05.22.2021>	#1 TS=(“asperger syndrome*” OR “autis*” OR “child development disorder*”)<br>#2 TS=(“mri” OR “magnetic resonance imaging” OR “smri” OR “s-mri” OR “structural- mri”)<br>#3 TS=(“artificial intelligence*” OR “deep learning*” OR “machine learning*” OR “machine intelligence*” OR “supervised learning*” OR “unsupervised learning*” OR “semi-supervised learning*” OR “reinforcement learning*” OR “regression algorithm*” OR “learning vector quantization*” OR “self organizing map*” OR “regularization algorithm*” OR “iterative dichotomiser*” OR “decision stump*” OR “clustering algorithm*” OR “k-means” OR “k-medians” OR “hierarchical clustering*” OR “apriori” OR “auto encoder*” OR “auto-encoder*” OR “markov” OR “classification algorithm*” OR “locally weighted learning*” OR “support vector machine*” OR “svm” OR ”decision tree*” OR “component analy*” OR “pca” OR “ica” OR “neural network*” OR “hierarchical learning*” OR “latent class analy*” OR “latent class model*” OR “latent variable model*” OR “boltzmann” OR “deep belief network*” OR “lasso regression*” OR “lda” OR “adtree*” OR “ridge regression*” OR “enet” OR “fnn” OR “firefly algorithm*” OR “multivariate adaptive regression splines*” OR “locally estimated scatterplot smoothing*” OR “instance base*” OR “instance-base*” OR “nearest neighbor*” OR “regularization algorithm*” OR “least absolute shrinkage select*” OR “least angle regression*” OR “eclat” OR “back propagation*” OR “hopfield network*” OR “radial basis function network*” OR “dimensionality reduction algorithm*” OR “ensemble algorithm*” OR “adaboost” OR “stacked generalization*” OR “relu” OR “naive bayes*”) #4 #1 AND #2 AND #3
**OpenGrey** <04.18.2021>	(“autis*” OR “asperger*” OR “child* development* disorder*”) AND ("mri" OR "magnetic resonance imaging" OR "structural mri" OR "smri" OR "s-mri")
**CRL** <1949 to 04.18.2021>	((autis*) or (asperger*) or (child development disorder*)) and ((mri) or (magnetic resonance imaging) or (structural mri) or (smri) or (s-mri))
**OATD** <04.18.2021>	title:(((autis*) OR ("asperger syndrom*") OR ("child development disorder*")) AND ((mri) OR (“magnetic resonance imaging”) OR (“structural mri”) OR (smri) OR (s-mri)) AND ((“artificial intelligence*”) OR (“deep learning*”) OR (“machine learning*”) OR (“machine intelligence*”) OR (“supervised learning*”) OR (“unsupervised learning*”) OR (“semi- supervised learning*”) OR (“reinforcement learning*”) OR (“regression algorithm*”) OR (“learning vector quantization*”) OR (“self organizing map*”) OR (“regularization algorithm*”) OR (“iterative dichotomiser*”) OR (“decision stump*”) OR (“clustering algorithm*”) OR (“k-means”) OR (“k-medians”) OR (“hierarchical clustering*”) OR (“apriori”) OR (“auto encoder*”) OR (“auto-encoder*”) OR (“markov”) OR (“classification algorithm*”) OR (“locally weighted learning*”) OR (“support vector machine*”) OR (“svm”) OR (”decision tree*”) OR (“component analy*”) OR (“pca”) OR (“ica”) OR (“neural network*”) OR (“hierarchical learning*”) OR (“latent class analy*”) OR (“latent class model*”) OR (“latent variable model*”) OR (“boltzmann”) OR (“deep belief network*”) OR (“lasso regression*”) OR (“lda”) OR (“adtree*”) OR (“ridge regression*”) OR (“enet”) OR (“fnn”) OR (“firefly algorithm*”) OR (“multivariate adaptive regression splines*”) OR (“locally estimated scatterplot smoothing*”) OR (“instance base*”) OR (“instance-base*”) OR (“nearest neighbor*”) OR (“regularization algorithm*”) OR (“least absolute shrinkage select*”) OR (“least angle regression*”) OR (“eclat”) OR (“back propagation*”) OR (“hopfield network*”) OR (“radial basis function network*”) OR (“dimensionality reduction algorithm*”) OR (“ensemble algorithm*”) OR (“adaboost”) OR (“stacked generalization*”) OR (“relu”) OR (“naive bayes*”)))

Rs-fMRI review

**Database** <date>	**Search syntax**
**Embase** Elsevier <1974 to 05.22.2021>	#1 ‘autism’/exp<br>#2 'asperger syndrom*':ab,ti,kw OR 'autis*':ab,ti,kw OR 'child* development* disorder*':ab,ti,kw<br>#3 #1 OR #2<br>#4 ‘functional magnetic resonance imaging’/exp<br>#5 fmri:ab,ti,kw OR 'functional magnetic resonance imag*':ab,ti,kw OR f-mri:ab,ti,kw OR 'functional mri':ab,ti,kw OR ‘functional-mri’:ab,ti,kw OR ‘functional mr imag*’:ab,ti,kw<br>#6 #4 OR #5<br>#7 ‘information processing’/exp<br>#8 'artificial intelligence*':ab,ti,kw OR 'deep learning*':ab,ti,kw OR 'machine learning*':ab,ti,kw OR 'machine intelligence*':ab,ti,kw OR 'supervised learning*':ab,ti,kw OR 'unsupervised learning*':ab,ti,kw OR ‘semi-supervised learning*’:ab,ti,kw OR ‘reinforcement learning*’:ab,ti,kw OR 'regression algorithm*':ab,ti,kw OR 'learning vector quantization*':ab,ti,kw OR 'self organizing map*':ab,ti,kw OR 'regularization algorithm*':ab,ti,kw OR 'iterative dichotomiser*':ab,ti,kw OR 'decision stump*':ab,ti,kw OR 'clustering algorithm*':ab,ti,kw OR 'k-means*':ab,ti,kw OR 'k-medians*':ab,ti,kw OR 'hierarchical clustering*':ab,ti,kw OR 'apriori*':ab,ti,kw OR 'auto encoder*':ab,ti,kw OR 'auto-encoder*':ab,ti,kw OR 'markov*':ab,ti,kw OR 'classification algorithm*':ab,ti,kw OR 'locally weighted learning*':ab,ti,kw OR 'support vector machine*':ab,ti,kw OR 'svm':ab,ti,kw OR 'decision tree*':ab,ti,kw OR 'component analy*':ab,ti,kw OR 'pca':ab,ti,kw OR 'ica':ab,ti,kw OR 'neural network*':ab,ti,kw OR 'hierarchical learning*':ab,ti,kw OR 'latent class analy*':ab,ti,kw OR 'latent class model*':ab,ti,kw OR 'latent variable model*':ab,ti,kw OR 'boltzmann':ab,ti,kw OR 'deep belief network*':ab,ti,kw OR ‘lasso regression*':ab,ti,kw OR ‘lda':ab,ti,kw OR ‘adtree':ab,ti,kw OR ‘ridge regression':ab,ti,kw OR ‘enet':ab,ti,kw OR ‘fnn':ab,ti,kw OR ‘firefly algorithm*':ab,ti,kw OR ‘multivariate adaptive regression splines*’:ab,ti,kw OR ‘locally estimated scatterplot smoothing*’:ab,ti,kw OR ‘instance base*’:ab,ti,kw OR ‘nearest neighbor*’:ab,ti,kw OR ‘regularization algorithm*’:ab,ti,kw OR ‘least absolute shrinkage select*’:ab,ti,kw OR ‘least angle regression*’:ab,ti,kw OR ‘eclat*’:ab,ti,kw OR ‘back propagation*’:ab,ti,kw OR ‘hopfield network*’:ab,ti,kw OR ‘radial basis function network*’:ab,ti,kw OR ‘dimensionality reduction algorithm*’:ab,ti,kw OR ‘ensemble algorithm*’:ab,ti,kw OR ‘adaboost’:ab,ti,kw OR ‘stacked generalization*’:ab,ti,kw OR ‘relu’:ab,ti,kw OR ‘naive bayes’:ab,ti,kw<br>#9 #7 OR #8<br>#10 #3 AND #6 AND #9
**MEDLINE** OvidSP <1996 to 05.22.2021>	1 exp Autism Spectrum Disorder/<br>2 (asperger syndrom* or autis* or child* development* disorder*).mp.<br>3 1 or 2<br>4 exp Magnetic Resonance Imaging/<br>5 (fmri or functional magnetic resonance imag* or f-mri or functional mri or functional- mri or functional mr imag*).mp.<br>6 4 or 5<br>7 exp Algorithms/ or exp Neural Networks, Computer/ or exp Decision Trees/<br>8 (artificial intelligence* or deep learning* or machine learning* or machine intelligence* or supervised learning* or unsupervised learning* or semi-supervised learning* or reinforcement learning* or regression algorithm* or learning vector quantization* or self organizing map* or regularization algorithm* or iterative dichotomiser* or decision stump* or clustering algorithm* or k-means* or k-medians* or hierarchical clustering* or apriori* or auto encoder* or auto-encoder* or markov* or classification algorithm* or locally weighted learning* or support vector machine* or svm or decision tree* or component analy* or pca or ica or neural network* or hierarchical learning* or latent class analy* or latent class model* or latent variable model* or boltzmann or deep belief network* or lasso regression* or lda or adtree or ridge regression or enet or fnn or firefly algorithm* or multivariate adaptive

	regression splines* or locally estimated scatterplot smoothing* or instance base* or nearest neighbor* or regularization algorithm* or least absolute shrinkage select* or least angle regression* or eclat* or back propagation* or hopfield network* or radial basis function network* or dimensionality reduction algorithm* or ensemble algorithm* or adaboost or stacked generalization* or relu or naive bayes).mp.<br>9 7 or 8<br>10 3 and 6 and 9
**APA PsycINFO** OvidSP <1987 to 05.22.2021>	1 exp Autism Spectrum Disorders/<br>2 (asperger syndrom* or autis* or child* development* disorder*).mp.<br>3 1 or 2<br>4 exp Functional Magnetic Resonance Imaging/<br>5 (fmri or functional magnetic resonance imag* or f-mri or functional mri or functional- mri or functional mr imag*).mp.<br>6 4 or 5<br>7 exp Artificial Intelligence/<br>8 (artificial intelligence* or deep learning* or machine learning* or machine intelligence* or supervised learning* or unsupervised learning* or semi-supervised learning* or reinforcement learning* or regression algorithm* or learning vector quantization* or self organizing map* or regularization algorithm* or iterative dichotomiser* or decision stump* or clustering algorithm* or k-means* or k-medians* or hierarchical clustering* or apriori* or auto encoder* or auto-encoder* or markov* or classification algorithm* or locally weighted learning* or support vector machine* or svm or decision tree* or component analy* or pca or ica or neural network* or hierarchical learning* or latent class analy* or latent class model* or latent variable model* or boltzmann or deep belief network* or lasso regression* or lda or adtree or ridge regression or enet or fnn or firefly algorithm* or multivariate adaptive regression splines* or locally estimated scatterplot smoothing* or instance base* or nearest neighbor* or regularization algorithm* or least absolute shrinkage select* or least angle regression* or eclat* or back propagation* or hopfield network* or radial basis function network* or dimensionality reduction algorithm* or ensemble algorithm* or adaboost or stacked generalization* or relu or naive bayes).mp.<br>9 7 or 8<br>10 3 and 6 and 9
**IEEE Xplore** <1988 to 05.22.2021>	(asperger syndrome OR autis* OR child development disorder) AND (fmri OR functional magnetic resonance imag* OR f-mri OR functional mri OR functional-mri OR functional mr imag*) AND (artificial intelligence OR deep learning OR machine learning OR machine intelligence OR supervised learning OR unsupervised learning OR semi- supervised learning OR reinforcement learning OR regression algorithm OR learning vector quantization OR self organizing map OR regularization algorithm OR iterative dichotomiser OR decision stump OR clustering algorithm OR k-means OR k-medians OR hierarchical clustering OR apriori OR auto encoder OR auto-encoder OR markov OR classification algorithm OR locally weighted learning OR support vector machine OR svm OR decision tree* OR component analysis OR pca OR ica OR neural network* OR hierarchical learning OR latent class analysis OR latent class model OR latent variable model OR boltzmann OR deep belief network OR lasso regression OR lda OR adtree OR ridge regression OR enet OR fnn OR firefly algorithm OR multivariate adaptive regression splines OR locally estimated scatterplot smoothing OR instance base OR nearest neighbors OR regularization algorithm OR least absolute shrinkage selection OR least angle regression OR eclat OR back propagation OR hopfield network OR radial basis function network OR dimensionality reduction algorithm OR ensemble algorithm OR adaboost OR stacked generalization OR relu OR naive bayes)
**Scopus** Elsevier <1996 to 05.22.2021>	TITLE-ABS-KEY("asperger syndrome*" OR "autis*" OR "child development disorder*") AND TITLE-ABS-KEY("fmri" OR "functional magnetic resonance imag*" OR "f-mri" OR "functional mri" OR "functional-mri" OR "functional mr imag*") AND TITLE-ABS- KEY("artificial intelligence*" OR "deep learning*" OR "machine learning*" OR "machine intelligence*" OR "supervised learning*" OR "unsupervised learning*" OR "semi- supervised learning*" OR "reinforcement learning*" OR "regression algorithm*" OR "learning vector quantization*" OR "self organizing map*" OR "regularization algorithm*" OR "iterative dichotomiser*" OR "decision stump*" OR "clustering algorithm*" OR "k- means" OR "k-medians" OR "hierarchical clustering*" OR "apriori" OR "auto encoder*" OR "auto-encoder*" OR "markov" OR "classification algorithm*" OR "locally weighted learning*" OR "support vector machine*" OR "svm" OR "decision tree*" OR "component analy*" OR "pca" OR "ica" OR "neural network*" OR "hierarchical learning*" OR "latent class analy*" OR "latent class model*" OR "latent variable model*" OR "boltzmann" OR "deep belief network*" OR "lasso regression*" OR "lda" OR "adtree*" OR "ridge regression*" OR "enet" OR "fnn" OR "firefly algorithm*" OR "multivariate adaptive regression splines*" OR "locally estimated scatterplot smoothing*" OR "instance base*"

	OR "instance-base*" OR "nearest neighbor*" OR "regularization algorithm*" OR "least absolute shrinkage select*" OR "least angle regression*" OR "eclat" OR "back propagation*" OR "hopfield network*" OR "radial basis function network*" OR "dimensionality reduction algorithm*" OR "ensemble algorithm*" OR "adaboost" OR "stacked generalization*" OR "relu" OR "naive bayes*")
**Web of Science** Core Collection <1900 to 05.22.2021>	#1 TS=("asperger syndrome*" OR "autis*" OR "child development disorder*")<br>#2 TS=("fmri" OR "functional magnetic resonance imag*" OR "f-mri" OR "functional mri" OR "functional-mri" OR "functional mr imag*")<br>#3 TS=("artificial intelligence*" OR "deep learning*" OR "machine learning*" OR "machine intelligence*" OR "supervised learning*" OR "unsupervised learning*" OR "semi-supervised learning*" OR "reinforcement learning*" OR "regression algorithm*" OR "learning vector quantization*" OR "self organizing map*" OR "regularization algorithm*" OR "iterative dichotomiser*" OR "decision stump*" OR "clustering algorithm*" OR "k-means" OR "k-medians" OR "hierarchical clustering*" OR "apriori" OR "auto encoder*" OR "auto-encoder*" OR "markov" OR "classification algorithm*" OR "locally weighted learning*" OR "support vector machine*" OR "svm" OR "decision tree*" OR "component analy*" OR "pca" OR "ica" OR "neural network*" OR "hierarchical learning*" OR "latent class analy*" OR "latent class model*" OR "latent variable model*" OR "boltzmann" OR "deep belief network*" OR "lasso regression*" OR "lda" OR "adtree*" OR "ridge regression*" OR "enet" OR "fnn" OR "firefly algorithm*" OR "multivariate adaptive regression splines*" OR "locally estimated scatterplot smoothing*" OR "instance base*" OR "instance-base*" OR "nearest neighbor*" OR "regularization algorithm*" OR "least absolute shrinkage select*" OR "least angle regression*" OR "eclat" OR "back propagation*" OR "hopfield network*" OR "radial basis function network*" OR "dimensionality reduction algorithm*" OR "ensemble algorithm*" OR "adaboost" OR "stacked generalization*" OR "relu" OR "naive bayes*") #4 #1 AND #2 AND #3
**OpenGrey** <04.18.2021>	("autis*" OR "asperger*" OR "child* development* disorder*") AND ("fmri" OR "functional magnetic resonance imag*" OR "f-mri" OR "functional mri" OR "functional-mri" OR "functional mr imag*")
**CRL** <1949 to 04.18.2021>	((autis*) or (asperger*) or (child development disorder*)) and ((fmri) or (functional magnetic resonance imag*) or (f-mri) or (functional mri) or (functional-mri) or (functional mr imag*))
**OATD** <04.18.2021>	title:(((autis*) OR ("asperger syndrom*") OR ("child development disorder*")) AND (("fmri") or ("functional magnetic resonance imag*") or ("f-mri") or ("functional mri") or ("functional-mri") or ("functional mr imag*")) AND (("artificial intelligence*") OR ("deep learning*") OR ("machine learning*") OR ("machine intelligence*") OR ("supervised learning*") OR ("unsupervised learning*") OR ("semi-supervised learning*") OR ("reinforcement learning*") OR ("regression algorithm*") OR ("learning vector quantization*") OR ("self organizing map*") OR ("regularization algorithm*") OR ("iterative dichotomiser*") OR ("decision stump*") OR ("clustering algorithm*") OR ("k- means") OR ("k-medians") OR ("hierarchical clustering*") OR ("apriori") OR ("auto encoder*") OR ("auto-encoder*") OR ("markov") OR ("classification algorithm*") OR ("locally weighted learning*") OR ("support vector machine*") OR ("svm") OR ("decision tree*") OR ("component analy*") OR ("pca") OR ("ica") OR ("neural network*") OR ("hierarchical learning*") OR ("latent class analy*") OR ("latent class model*") OR ("latent variable model*") OR ("boltzmann") OR ("deep belief network*") OR ("lasso regression*") OR ("lda") OR ("adtree*") OR ("ridge regression*") OR ("enet") OR ("fnn") OR ("firefly algorithm*") OR ("multivariate adaptive regression splines*") OR ("locally estimated scatterplot smoothing*") OR ("instance base*") OR ("instance-base*") OR ("nearest neighbor*") OR ("regularization algorithm*") OR ("least absolute shrinkage select*") OR ("least angle regression*") OR ("eclat") OR ("back propagation*") OR ("hopfield network*") OR ("radial basis function network*") OR ("dimensionality reduction algorithm*") OR ("ensemble algorithm*") OR ("adaboost") OR ("stacked generalization*") OR ("relu") OR ("naive bayes*")))

EEG review

Database <date>	Search syntax
**Embase** Elsevier <1974 to 05.22.2021>	#1 'autism'/exp<br>#2 'asperger syndrom*':ab,ti,kw OR 'autis*':ab,ti,kw OR 'child* development* disorder*':ab,ti,kw<br>#3 #1 OR #2<br>#4 'electroencephalogram'/exp<br>#5 'electroencephalogra*':ti,ab,kw OR 'eeg':ti,ab,kw OR 'alpha rhythm*':ti,ab,kw OR 'beta rhythm*':ti,ab,kw OR 'delta rhythm*':ti,ab,kw OR 'gamma rhythm*':ti,ab,kw OR 'theta rhythm*':ti,ab,kw OR 'mu rhythm*':ti,ab,kw OR 'brain wave*':ti,ab,kw OR 'brain electric* activit*':ab,ti,kw OR 'brainwave*':ab,ti,kw<br>#6 #4 OR #5<br>#7 'information processing'/exp<br>#8 'artificial intelligence*':ab,ti,kw OR 'deep learning*':ab,ti,kw OR 'machine learning*':ab,ti,kw OR 'machine intelligence*':ab,ti,kw OR 'supervised learning*':ab,ti,kw OR 'unsupervised learning*':ab,ti,kw OR 'semi-supervised learning*':ab,ti,kw OR 'reinforcement learning*':ab,ti,kw OR 'regression algorithm*':ab,ti,kw OR 'learning vector quantization*':ab,ti,kw OR 'self organizing map*':ab,ti,kw OR 'regularization algorithm*':ab,ti,kw OR 'iterative dichotomiser*':ab,ti,kw OR 'decision stump*':ab,ti,kw OR 'clustering algorithm*':ab,ti,kw OR 'k-means*':ab,ti,kw OR 'k-medians*':ab,ti,kw OR 'hierarchical clustering*':ab,ti,kw OR 'apriori*':ab,ti,kw OR 'auto encoder*':ab,ti,kw OR 'auto-encoder*':ab,ti,kw OR 'markov*':ab,ti,kw OR 'classification algorithm*':ab,ti,kw OR 'locally weighted learning*':ab,ti,kw OR 'support vector machine*':ab,ti,kw OR 'svm':ab,ti,kw OR 'decision tree*':ab,ti,kw OR 'component analy*':ab,ti,kw OR 'pca':ab,ti,kw OR 'ica':ab,ti,kw OR 'neural network*':ab,ti,kw OR 'hierarchical learning*':ab,ti,kw OR 'latent class analy*':ab,ti,kw OR 'latent class model*':ab,ti,kw OR 'latent variable model*':ab,ti,kw OR 'boltzmann':ab,ti,kw OR 'deep belief network*':ab,ti,kw OR 'lasso regression*':ab,ti,kw OR 'lda':ab,ti,kw OR 'adtree':ab,ti,kw OR 'ridge regression':ab,ti,kw OR 'enet':ab,ti,kw OR 'fnn':ab,ti,kw OR 'firefly algorithm*':ab,ti,kw OR 'multivariate adaptive regression splines*':ab,ti,kw OR 'locally estimated scatterplot smoothing*':ab,ti,kw OR 'instance base*':ab,ti,kw OR 'nearest neighbor*':ab,ti,kw OR 'regularization algorithm*':ab,ti,kw OR 'least absolute shrinkage select*':ab,ti,kw OR 'least angle regression*':ab,ti,kw OR 'eclat*':ab,ti,kw OR 'back propagation*':ab,ti,kw OR 'hopfield network*':ab,ti,kw OR 'radial basis function network*':ab,ti,kw OR 'dimensionality reduction algorithm*':ab,ti,kw OR 'ensemble algorithm*':ab,ti,kw OR 'adaboost':ab,ti,kw OR 'stacked generalization*':ab,ti,kw OR 'relu':ab,ti,kw OR 'naive bayes':ab,ti,kw<br>#9 #7 OR #8<br>#10 #3 AND #6 AND #9
**MEDLINE** OvidSP <1996 to 05.22.2021>	1 exp Autism Spectrum Disorder/<br>2 (asperger syndrom* or autis* or child* development* disorder*).mp.<br>3 1 or 2<br>4 exp Electroencephalography/<br>5 (electroencephalogra* or eeg or alpha rhythm* or beta rhythm* or delta rhythm* or gamma rhythm* or theta rhythm* or mu rhythm* or brain wave* or brain electric* activit* or brainwave*).mp.<br>6 4 or 5<br>7 exp Algorithms/ or exp Neural Networks, Computer/ or exp Decision Trees/<br>8 (artificial intelligence* or deep learning* or machine learning* or machine intelligence* or supervised learning* or unsupervised learning* or semi-supervised learning* or reinforcement learning* or regression algorithm* or learning vector quantization* or self organizing map* or regularization algorithm* or iterative dichotomiser* or decision stump* or clustering algorithm* or k-means* or k-medians* or hierarchical clustering* or apriori* or auto encoder* or auto-encoder* or markov* or classification algorithm* or locally weighted learning* or support vector machine* or svm or decision tree* or

	component analy* or pca or ica or neural network* or hierarchical learning* or latent class analy* or latent class model* or latent variable model* or boltzmann or deep belief network* or lasso regression* or lda or adtree or ridge regression or enet or fnn or firefly algorithm* or multivariate adaptive regression splines* or locally estimated scatterplot smoothing* or instance base* or nearest neighbor* or regularization algorithm* or least absolute shrinkage select* or least angle regression* or eclat* or back propagation* or hopfield network* or radial basis function network* or dimensionality reduction algorithm* or ensemble algorithm* or adaboost or stacked generalization* or relu or naive bayes).mp.<br>9 7 or 8<br>10 3 and 6 and 9
**APA PsycINFO** OvidSP <1987 to 05.22.2021>	1 exp Autism Spectrum Disorders/<br>2 (asperger syndrom* or autis* or child* development* disorder*).mp.<br>3 1 or 2<br>4 exp Electroencephalography/<br>5 (electroencephalogra* or eeg or alpha rhythm* or beta rhythm* or delta rhythm* or gamma rhythm* or theta rhythm* or mu rhythm* or brain wave* or brain electric* activit* or brainwave*).mp.<br>6 4 or 5<br>7 exp Artificial Intelligence/<br>8 (artificial intelligence* or deep learning* or machine learning* or machine intelligence* or supervised learning* or unsupervised learning* or semi-supervised learning* or reinforcement learning* or regression algorithm* or learning vector quantization* or self organizing map* or regularization algorithm* or iterative dichotomiser* or decision stump* or clustering algorithm* or k-means* or k-medians* or hierarchical clustering* or apriori* or auto encoder* or auto-encoder* or markov* or classification algorithm* or locally weighted learning* or support vector machine* or svm or decision tree* or component analy* or pca or ica or neural network* or hierarchical learning* or latent class analy* or latent class model* or latent variable model* or boltzmann or deep belief network* or lasso regression* or lda or adtree or ridge regression or enet or fnn or firefly algorithm* or multivariate adaptive regression splines* or locally estimated scatterplot smoothing* or instance base* or nearest neighbor* or regularization algorithm* or least absolute shrinkage select* or least angle regression* or eclat* or back propagation* or hopfield network* or radial basis function network* or dimensionality reduction algorithm* or ensemble algorithm* or adaboost or stacked generalization* or relu or naive bayes).mp.<br>9 7 or 8<br>10 3 and 6 and 9
**IEEE Xplore** <1988 to 05.22.2021>	(asperger syndrome OR autis* OR child development disorder) AND (electroencephalogra* OR eeg OR alpha rhythm* OR beta rhythm* OR delta rhythm* OR gamma rhythm OR theta rhythm OR mu rhythm OR brain wave OR brain electrical OR brain electrics OR brain electricals OR brain activity OR brainwave) AND (artificial intelligence OR deep learning OR machine learning OR machine intelligence OR supervised learning OR unsupervised learning OR semi-supervised learning OR reinforcement learning OR regression algorithm OR learning vector quantization OR self organizing map OR regularization algorithm OR iterative dichotomiser OR decision stump OR clustering algorithm OR k-means OR k-medians OR hierarchical clustering OR apriori OR auto encoder OR auto-encoder OR markov OR classification algorithm OR locally weighted learning OR support vector machine OR svm OR decision tree* OR component analysis OR pca OR ica OR neural network* OR hierarchical learning OR latent class analysis OR latent class model OR latent variable model OR boltzmann OR deep belief network OR lasso regression OR lda OR adtree OR ridge regression OR enet OR fnn OR firefly algorithm OR multivariate adaptive regression splines OR locally estimated scatterplot smoothing OR instance base OR nearest neighbors OR regularization algorithm OR least absolute shrinkage selection OR least angle regression OR eclat OR back propagation OR hopfield network OR radial basis function network OR dimensionality reduction algorithm OR ensemble algorithm OR adaboost OR stacked generalization OR relu OR naive bayes)
**Scopus** Elsevier <1996 to 05.22.2021>	TITLE-ABS-KEY(“asperger syndrome*” OR “autis*” OR “child development disorder*”) AND TITLE-ABS-KEY(“electroencephalogra*“ OR “eeg“ OR “alpha rhythm*“ OR “beta rhythm*“ OR “delta rhythm*“ OR “gamma rhythm*“ OR “theta rhythm*“ OR “mu rhythm*“ OR “brain wave*“ OR “brain electric*“ OR “brain activit*“ OR “brainwave*”) AND TITLE- ABS-KEY(“artificial intelligence*” OR “deep learning*” OR “machine learning*” OR “machine intelligence*” OR “supervised learning*” OR “unsupervised learning*” OR “semi-supervised learning*” OR “reinforcement learning*” OR “regression algorithm*” OR “learning vector quantization*” OR “self organizing map*” OR “regularization algorithm*” OR “iterative dichotomiser*” OR “decision stump*” OR “clustering

	algorithm*” OR “k-means” OR “k-medians” OR “hierarchical clustering*” OR “apriori” OR “auto encoder*” OR “auto-encoder*” OR “markov” OR “classification algorithm*” OR “locally weighted learning*” OR “support vector machine*” OR “svm” OR ”decision tree*” OR “component analy*” OR “pca” OR “ica” OR “neural network*” OR “hierarchical learning*” OR “latent class analy*” OR “latent class model*” OR “latent variable model*” OR “boltzmann” OR “deep belief network*” OR “lasso regression*” OR “lda” OR “adtree*” OR “ridge regression*” OR “enet” OR “fnn” OR “firefly algorithm*” OR “multivariate adaptive regression splines*” OR “locally estimated scatterplot smoothing*” OR “instance base*” OR “instance-base*” OR “nearest neighbor*” OR “regularization algorithm*” OR “least absolute shrinkage select*” OR “least angle regression*” OR “eclat” OR “back propagation*” OR “hopfield network*” OR “radial basis function network*” OR “dimensionality reduction algorithm*” OR “ensemble algorithm*” OR “adaboost” OR “stacked generalization*” OR “relu” OR “naive bayes*”)
**Web of Science** Core Collection <1900 to 05.22.2021>	#1 TS=(“asperger syndrome*” OR “autis*” OR “child development disorder*”)<br>#2 TS=(“electroencephalogra*“ OR “eeg“ OR “alpha rhythm*“ OR “beta rhythm*“ OR “delta rhythm*“ OR “gamma rhythm*“ OR “theta rhythm*“ OR “mu rhythm*“ OR “brain wave*“ OR “brain electric*“ OR “brain activit*“ OR “brainwave*”)<br>#3 TS=(“artificial intelligence*” OR “deep learning*” OR “machine learning*” OR “machine intelligence*” OR “supervised learning*” OR “unsupervised learning*” OR “semi-supervised learning*” OR “reinforcement learning*” OR “regression algorithm*” OR “learning vector quantization*” OR “self organizing map*” OR “regularization algorithm*” OR “iterative dichotomiser*” OR “decision stump*” OR “clustering algorithm*” OR “k-means” OR “k-medians” OR “hierarchical clustering*” OR “apriori” OR “auto encoder*” OR “auto-encoder*” OR “markov” OR “classification algorithm*” OR “locally weighted learning*” OR “support vector machine*” OR “svm” OR ”decision tree*” OR “component analy*” OR “pca” OR “ica” OR “neural network*” OR “hierarchical learning*” OR “latent class analy*” OR “latent class model*” OR “latent variable model*” OR “boltzmann” OR “deep belief network*” OR “lasso regression*” OR “lda” OR “adtree*” OR “ridge regression*” OR “enet” OR “fnn” OR “firefly algorithm*” OR “multivariate adaptive regression splines*” OR “locally estimated scatterplot smoothing*” OR “instance base*” OR “instance-base*” OR “nearest neighbor*” OR “regularization algorithm*” OR “least absolute shrinkage select*” OR “least angle regression*” OR “eclat” OR “back propagation*” OR “hopfield network*” OR “radial basis function network*” OR “dimensionality reduction algorithm*” OR “ensemble algorithm*” OR “adaboost” OR “stacked generalization*” OR “relu” OR “naive bayes*”) #4 #1 AND #2 AND #3
**OpenGrey** <04.18.2021>	(“autis*” OR “asperger*” OR “child* development* disorder*”) AND (“electroencephalogra*“ OR “eeg“ OR “alpha rhythm*“ OR “beta rhythm*“ OR “delta rhythm*“ OR “gamma rhythm*“ OR “theta rhythm*“ OR “mu rhythm*“ OR “brain wave*“ OR “brain electric*“ OR “brain activit*“ OR “brainwave*”) AND (“artificial intelligence*” OR “deep learning*” OR “machine learning*” OR “machine intelligence*” OR “supervised learning*” OR “unsupervised learning*” OR “semi-supervised learning*” OR “reinforcement learning*” OR “regression algorithm*” OR “learning vector quantization*” OR “self organizing map*” OR “regularization algorithm*” OR “iterative dichotomiser*” OR “decision stump*” OR “clustering algorithm*” OR “k-means” OR “k-medians” OR “hierarchical clustering*” OR “apriori” OR “auto encoder*” OR “auto-encoder*” OR “markov” OR “classification algorithm*” OR “locally weighted learning*” OR “support vector machine*” OR “svm” OR ”decision tree*” OR “component analy*” OR “pca” OR “ica” OR “neural network*” OR “hierarchical learning*” OR “latent class analy*” OR “latent class model*” OR “latent variable model*” OR “boltzmann” OR “deep belief network*” OR “lasso regression*” OR “lda” OR “adtree*” OR “ridge regression*” OR “enet” OR “fnn” OR “firefly algorithm*” OR “multivariate adaptive regression splines*” OR “locally estimated scatterplot smoothing*” OR “instance base*” OR “instance-base*” OR “nearest neighbor*” OR “regularization algorithm*” OR “least absolute shrinkage select*” OR “least angle regression*” OR “eclat” OR “back propagation*” OR “hopfield network*” OR “radial basis function network*” OR “dimensionality reduction algorithm*” OR “ensemble algorithm*” OR “adaboost” OR “stacked generalization*” OR “relu” OR “naive bayes*”)
**CRL** <1949 to 04.18.2021>	((autis*) or (asperger*) or (child development disorder*)) and ((electroencephalogra*) or (eeg) or (alpha rhythm*) or (beta rhythm*) or (delta rhythm*) or (gamma rhythm*) or (theta rhythm*) or (mu rhythm*) or (brain wave*) or (brain electric* activit*) or (brainwave*))
**OATD** <04.18.2021>	title:(((autis*) OR ("asperger syndrom*") OR ("child development disorder*")) AND ((electroencephalogra*) OR (eeg) OR (alpha rhythm*) OR (beta rhythm*) OR (delta rhythm*) OR (gamma rhythm*) OR (theta rhythm*) OR (mu rhythm*) OR (brain wave*) OR (brain electric* activit*) OR (brainwave*)) AND ((“artificial intelligence*”) OR (“deep learning*”) OR (“machine learning*”) OR (“machine intelligence*”) OR (“supervised learning*”) OR (“unsupervised learning*”) OR (“semi-supervised learning*”) OR (“reinforcement learning*”) OR (“regression algorithm*”) OR (“learning vector quantization*”) OR (“self organizing map*”) OR (“regularization algorithm*”) OR (“iterative dichotomiser*”) OR (“decision stump*”) OR (“clustering algorithm*”) OR (“k- means”) OR (“k-medians”) OR (“hierarchical clustering*”) OR (“apriori”) OR (“auto encoder*”) OR (“auto-encoder*”) OR (”markov”) OR (“classification algorithm*”) OR

	(“locally weighted learning*”) OR (“support vector machine*”) OR (“svm”) OR (”decision tree*”) OR (“component analy*”) OR (“pca”) OR (“ica”) OR (“neural network*”) OR (“hierarchical learning*”) OR (“latent class analy*”) OR (“latent class model*”) OR (“latent variable model*”) OR (“boltzmann”) OR (“deep belief network*”) OR (“lasso regression*”) OR (“lda”) OR (“adtree*”) OR (“ridge regression*”) OR (“enet”) OR (“fnn”) OR (“firefly algorithm*”) OR (“multivariate adaptive regression splines*”) OR (“locally estimated scatterplot smoothing*”) OR (“instance base*”) OR (“instance-base*”) OR (“nearest neighbor*”) OR (“regularization algorithm*”) OR (“least absolute shrinkage select*”) OR (“least angle regression*”) OR (“eclat”) OR (“back propagation*”) OR (“hopfield network*”) OR (“radial basis function network*”) OR (“dimensionality reduction algorithm*”) OR (“ensemble algorithm*”) OR (“adaboost”) OR (“stacked generalization*”) OR (“relu”) OR (“naive bayes*”)))
